# Feasibility and Effectiveness of a Novel Intervention Integrating Physical Therapy Exercise and Dance Movement Therapy on Fall Risk in Community-Dwelling Older Women: A Randomized Pilot Study

**DOI:** 10.3390/healthcare11081104

**Published:** 2023-04-12

**Authors:** Michal Pitluk Barash, Einat Shuper Engelhard, Michal Elboim-Gabyzon

**Affiliations:** 1 The Graduate School of Creative Art Therapies, Faculty of Social Welfare & Health Sciences, Emili Sagol Creative Arts Therapies Research Center, University of Haifa, Haifa 3498838, Israel; einat.shuper@gmail.com; 2 The Graduate School of Creative Art Therapies, Faculty of Humanities & Social Sciences, Kibbutzim College of Education, Tel Aviv 6250769, Israel; 3 Physical Therapy Department, Faculty of Social Welfare & Health Sciences, University of Haifa, Haifa 3498838, Israel; michal.elboim@gmail.com

**Keywords:** falls, fall risk, older adults, balance, fear of falling, fall-efficacy, home exercise adherence, physical therapy, dance movement therapy

## Abstract

This pilot study presents a novel fall prevention intervention that integrates physical therapy exercise (PTE) and dance movement therapy (DMT) to address both physical and emotional fall risk factors, as well as factors influencing adherence to treatment. The aim of this study was to examine the feasibility and effectiveness of the intervention in a sample of eight older women (median = 86 [81.25–90.75] years) from a day center for senior citizens. The intervention, based on the Otago Exercise Program and DMT techniques, aimed to address the emotional experience during physical exercise. Participants were randomly assigned to either a PTE+DMT intervention group (*n* = 5) or a PTE control group (*n* = 3). A pre–post intervention battery of physical and emotional fall risk assessments, therapist–patient bond, and home exercise adherence was conducted. Non-parametric tests results showed significant improvement in the PTE+DMT group in measures of balance and fear of falling compared to the PTE group. However, no other significant differences were found between the groups in terms of falls-related psychological concerns, self-perceived health status, therapist–patient bond, and home exercise adherence. These findings demonstrate the feasibility and potential benefits of an intervention that integrates both physical and emotional aspects to reduce fall risk in older adults, and provide a basis for further studies and modifications in the research protocol.

## 1. Introduction

Falls are the second leading cause of unintentional mortality worldwide and a major cause of morbidity among the older adult population [1]. Globally, every year, 30% of community-dwelling adults aged 65 years and over fall at least once [2] and 10% fall at least twice [3]. The risk of falls increases with age due to changes in health status related to physical, sensory, and cognitive decline [4,5,6]. In addition, decreased functional ability in activities connected to daily living (ADL) [7,8], and reduced levels of physical activity increase the risk of falling [9].

Physical inactivity amplifies the fall risk factors associated with aging, such as decreased balance and muscle strength [10]. According to a review by The Cochrane Library, physical exercises aimed at improving balance are crucial components of fall prevention programs and can reduce the rate of falls in older adults by approximately 23% [11]. The Otago Exercise Program (OEP) is a widely accepted physical therapy (PT) program for fall prevention that has been proven effective. It consists of muscle strength and balance exercises, walking, and ongoing home practice [12]. Although originally designed for independent practice by community-dwelling older adults, the OEP can also be used in group settings [13]. However, studies have shown that addressing all the risk factors for falls, including emotional and social factors, is essential to increase the effectiveness of fall prevention programs, rather than focusing solely on the physical aspect [14,15]. To date, these factors have not been examined as part of an integrative intervention for fall prevention in older adults.

Emotional state has a significant impact on the risk of falls, as well as the ability to cope with the aftermath of a fall [16,17,18]. In addition, emotional state affects risk factors such as health status and functional ability. Falls-related psychological concerns, including fear of falling (FOF) and falls-efficacy (FSe), have been found to increase the risk of falls [16,19]. FOF is a common psychological concern among older adults, affecting approximately a third of those who have fallen and nearly 50% of older adults who have not experienced a fall [20]. FOF is associated with negative health outcomes such as decreased physical and cognitive functioning, decreased mental health and well-being [21,22], increased symptoms of depression, frequent experiences of loneliness [23], and poor quality of life [24]. FOF can also result in limitations in physical activity related to daily life [25] and avoidance of physical and social activities [21], even in individuals without a history of falls [26]. FSe, which refers to an individual’s confidence in performing daily activities without falling, is another emotional factor involved in falls. Higher levels of FSe have been associated with fewer falls [27], a higher level of quality of life in terms of physical and mental components [28], and better balance [29]. FSe has also been found to reduce FOF, leading to fewer limitations in ADL and increased functional capacity [30].

Adherence to physical activity, which is essential for preventing falls in older adults, may be challenging to maintain due to its decline over time. In addition, physical and emotional factors such as health and functional conditions, FOF, and self-efficacy decrease adherence to physical activity regimes [31,32,33,34,35,36]. The therapist–patient relationship can help increase adherence to physical exercise aimed at preventing falls. Factors such as establishing a secure and trustworthy relationship, encouraging cooperation and reciprocity, offering emotional support, showing empathy, expressing interest in patients’ lives, and understanding and addressing their needs through verbal and non-verbal means can all contribute to increased persistence in treatment [37,38].

Thus, overall, this literature suggests that the emotional aspect is a significant factor that affects the risk of falling and adherence to fall prevention programs, and can increase potential physical risk factors. This points to the need for integrated treatment that includes physical interventions, such as PT, with emotional-physical therapy to reduce the risk of falls and their associated negative consequences. Dance movement therapy (DMT) utilizes emotional, physical, cognitive, and social integration to engender an emotional response to a physical-emotional experience that can improve health and mental well-being [39]. Previous studies have shown that DMT decreases depression, anxiety, and negative mood, and increases quality of life [40,41]. In DMT, the grounding technique, which focuses on stability and balance, is based on a psychosomatic model in which physical and emotional processes are intertwined, based on the assumption that interventions to improve grounding can change both breathing and posture as well as emotional awareness and emotional regulation [42,43,44]. Through inviting movement and utilizing proprioception, balance, and the tactile system, DMT enables the processing of emotional experiences that arise during movement. This is facilitated by an emotional, empathetic therapist who is attuned to the patient and their relationship [45]. The utilization of grounding techniques increases awareness of physical abilities, strengthens sensory perception and the sense of security [46], enhances sensory awareness, and contributes to mental relaxation [47]. Studies have also found that this technique can improve body awareness, somatic sensations, and mental experiences [48]. Clinical cases have highlighted the importance of group work in improving grounding, fostering mutual contact among group members, promoting social empowerment, and reducing feelings of loneliness [46].

Fall prevention programs have traditionally focused on the physical aspect of falling in older adults, and have not adequately examined the role of the emotional aspect in preventing falls as a means of enhancing the effectiveness of the program and treatment adherence. This literature review suggests that integrating physical intervention to prevent falls from PT with the emotional responses elicited in DMT can address both physical and emotional fall risk factors and treatment adherence. The aim of the current study was to investigate the feasibility and effectiveness of a novel group intervention integrating PT exercises and DMT (PTE+DMT) in reducing the risk of falls in older adults. Our hypothesis was that the PTE+DMT group would show greater improvement in (1) health-physical factors that affect falling (fall risk, functioning in ADL, objective balance, and self-perceived health status); (2) emotional factors that affect falling (FOF, FSe, balance confidence, and FOF avoidance behavior); and (3) factors influencing adherence to treatment (therapist–patient bond and home exercise adherence), compared to the PTE group.

## 2. Materials and Methods

### 2.1. Study Design

This quantitative pilot study is part of a research project aiming to construct and assess a novel intervention model for preventing falls in older adults that integrates PTE and DMT. The current study will be followed by a larger randomized controlled trial (RCT). This pilot study employed a two-group, parallel RCT design. The trial was reported following the Consolidated Standards of Reporting Trials (CONSORT) guidelines [49]. The study was approved by the ethics committee of the University of Haifa, Haifa, Israel (Approval 2699).

### 2.2. Participants

Eighteen participants were recruited for the study at a day center for senior citizens through convenience sampling by a social worker who was not involved in the intervention process. Of the initial 18 participants who agreed to participate, 10 participants were found to be suitable for the study according to the eligibility criteria. None of the participants dropped out during the intervention sessions. Of the 10 participants who participated in the intervention, two were excluded from the data analysis because they did not participate in the post-intervention assessments.

#### 2.2.1. Eligibility Criteria

Participants were thoroughly screened for eligibility by two physical therapists and the social worker at the day center. The inclusion criteria were: (1) community-dwelling older adults aged 65 or over; (2) the ability to walk independently for at least 10 m with or without an assistive device; and (3) the ability to walk for 2 min without an assistive device. Exclusion criteria included: (1) heart disease or respiratory disease; (2) a history of stroke, Parkinson’s, or any other neurological disorder affecting walking; (3) blindness or deafness that would prevent safe walking and hearing instructions; (4) a vestibular disorder such as vertigo; (5) acute joint pain in the lower limbs that would limit continuous walking for 2 min; and (6) significant sensation problems in the lower limbs.

#### 2.2.2. Random Assignment

The social worker at the day center employed stratified randomization to allocate eligible participants to one of the two groups, in order to ensure an approximate in mobility ability (i.e., assistive device use) among individuals allocated to each group. In the PTE+DMT group, there were six participants, and in the PTE control group there were five participants.

### 2.3. Interventions- PTE+DMT and PTE

The interventions took place in a 40 m^2^ (430.5 square feet) room suitable for movement, in the day center. Both groups participated in an intervention based on OEP [12,50]. Each group took part in six group sessions lasting 40 min, twice a week for 3 weeks.

The intervention included a structured set of exercises increasing in difficulty from week-to-week: a 5 min warm-up, 10 min strength exercises, 20 min balance exercises, and a 5 min cool-down. The PTE+DMT group was given an intervention that integrated OEP-based techniques and DMT techniques to address the emotional experience that arises during physical exercise. This included encouragement for spontaneous and creative movement on the part of the participants, and an invitation to verbally share the feelings, sensations, and thoughts that arose in the participants following the movement. For example, during an exercise in which participants raised to the tips of their toes from a standing position, the instructor asked them to pay attention to the space between their legs, and to consider what helps them feel stable, as well as what constitutes a safe foundation in life for them. The group was led by a dance movement therapist who was supervised by an expert in the field with over 20 years of experience. The therapist had received training in OEP. The PTE control group received only an OEP-based intervention, which was led by a physical therapist who was supervised by an expert in the field with over 20 years of experience. Both instructors were present in sessions of both groups and were responsible for ensuring the safety of the participants. To ensure the fidelity of the interventions, the instructors went through the sequence of exercises together before each session. Additionally, the instructors received the same number of supervisory hours.

### 2.4. Measurements

#### 2.4.1. Participant Characteristics

Self-report questionnaire regarding demographic status and clinical data which included information such as age, marital status, medications, and background illnesses.

#### 2.4.2. Fall Risk

Fall risk assessment was performed using the Fall Risk Self-Assessment Tool (FRQ), which is a self-report questionnaire that includes 12 items rated on a dichotomous scale of “yes” or “no” [51]. The overall score of the questionnaire is the sum of the items to which the answer is “yes”, and the score range is between 0 and 14. A score of 4 or higher indicates a level of risk of falling that requires a visit to a healthcare provider. The English version of the FRQ has been found to be reliable and valid, (α = 0.746; Kappa = 0.875, *p* < 0.0001).

#### 2.4.3. Functioning in ADL

An assessment of Functioning in ADL was performed using the Barthel Index, which is a self-report questionnaire that includes 10 items rated on a 2- or 3-point ordinal scale that reflects the degree of independence of the individual in performing each ADL, and the degree of mobility independence [52]. The overall score is the sum of the items and score range is between 0 and 100. A score of 0 indicates complete dependence and a score of 100 indicates complete independence. The English version of the Barthel Index has been found to be reliable (ICC = 0.87–0.92; Kendall’s coefficient = 0.93) [53].

#### 2.4.4. Objective Balance

Objective balance assessment was performed using the Timed Up and Go (TUG) [54] and the Five Times Sit to Stand (5STS) tests [55]. In the TUG test, the time taken by participants while rising from a chair (46 cm height), walking 3 m, turning, walking back, and sitting down, was measured. The TUG is conducted twice, and the average of the two test scores determines the overall score. A score higher than 13.5 s indicates a risk of falling among community-dwelling older adults [56]. The TUG has been found to be reliable and valid (ICC = 0.99; *r* = −0.81) [54].

In the 5STS test, the time taken by participants while getting up from a seated position in a chair and standing up and sitting down five times, was measured. Norm scores for ages 70 to 79 and 80 to 89 are 12.6 s and 14.8 s, respectively [57]. The 5STS has been found to be reliable (ICC = 0.957) [58], and valid (*r* = 0.918) [59].

#### 2.4.5. FOF

FOF assessment was performed using the FOF single item question (FOF SIQ) [60] and the Fear of Falling Questionnaire Revised (FFQ-R) [61]. The FOF SIQ, is a single self-report question: “Are you afraid of falling?”. The question is rated on a Likert-type scale ranging from 1 (*no, not afraid*) to 4 (*yes, indeed afraid*). The English version of the question has been found to be valid (*r* = −0.59).

The FFQ-R is a self-report questionnaire that includes six items rated on Likert-type scale ranging from 1 (*strongly disagree*) to 4 (*strongly agree*). The overall score is the sum of the items and score range is between 6 and 24. A higher scores indicate higher FOF levels. The English version of the question has been found to be reliable and valid (α = 0.957; *r* = 0.42).

#### 2.4.6. FSe

FSe assessment was performed using the Short Falls Efficacy Scale-International (Short FES-I), which is a self-report questionnaire that includes seven items rated on a Likert-type scale ranging from 1 (*not at all concerned*) to 4 (*extremely concerned*) [62]. The overall score of the questionnaire is the sum of its items, and the score range is between 7 and 28. A lower score indicates better self-efficacy related to falls. The English version of the questionnaire has been found to be reliable and valid (α = 0.92; ρ = 0.97).

#### 2.4.7. Balance Confidence

Balance Confidence assessment was performed using the Activities-Specific Balance Confidence (ABC) scale, which is a self-report questionnaire that includes 16 items rated on a scale ranging from 0% (*no confidence*) to 100% (*complete confidence*) [63]. Participants rate their perceived ability to perform different activities ranging from walking around the house or reaching for an object at shoulder level, to walking outside on an icy sidewalk. The overall score of the questionnaire is the items average, and the score range is between 0 and 100. A higher score indicates greater confidence in performing ADL. The English version of the questionnaire has been found to be reliable and valid (α = 0.96; *r* = 0.63) [20]. The Hebrew version of the questionnaire has been found to be reliable and valid (α = 0.96; *r* = 0.542) [64].

#### 2.4.8. FOF Avoidance Behavior

FOF avoidance behavior assessment was performed using the Fear of Falling Avoidance Behavior Questionnaire (FFABQ), which is a self-report questionnaire that includes 14 items rated on a Likert-type scale ranging from 0 (*completely disagree*) to 4 (*completely agree*) [65]. Participants rate their self-imposed avoidance of activities due to FOF. The overall score of the questionnaire is the sum of its items, and the score range is between 0 and 56. A higher score indicates greater activity limitation and participation restriction as a result of the FOF. The English version of the questionnaire has been found to be reliable and valid (ICC = 0.812; *r* = 0.678).

#### 2.4.9. Self-Perceived Health Status

Self-perceived health status assessment was performed using the 12-item Short Form Health Survey (SF-12), which is a self-report questionnaire that includes 12 items that are summarized as two scores: the Physical Component Summary (PCS-12), which refers to the physical aspect of health status; and the Mental Component Summary (MCS-12), which refers to the mental aspect of health status [66]. Four items are rated on a dichotomous yes/no scale, while the remaining items are rated on a Likert-type scale with response options ranging from 3 to 6. In calculating the PCS-12 and MCS-12 scores, each item contributes to each score according to a predetermined weight based on the general population in the United States. A study that examined the questionnaire in nine languages found that it is recommended to use the summary scores derived from the population in the United States [67]. The average score in each component is 50, and the standard deviation is 10. The overall scores range from 0 to 100. A higher score indicates a better perception of health status and quality of life. The English version of the questionnaire has been found to be reliable and valid [66]. Additionally, it has been translated into Hebrew using the standard procedure recommended by the developers of the questionnaire [68] and was found to be reliable and valid among community-dwelling older adults (α = 0.71–0.86; *r* = 0.61–0.68) [69].

#### 2.4.10. Therapist–Patient Bond

Therapist–patient bond assessment was performed using the Therapeutic Bond subscale of the 36-item Working Alliance Inventory [70]. The therapeutic bond subscale is a self-report questionnaire that includes 12 items rated by the patient on a Likert-type scale ranging from 1 (*does not describe my feeling at all*) to 5 (*very much describes my feeling*). For example, “My relationship with my therapist is very important to me.” The overall score of the questionnaire is the items average, and the score range is between 1 and 5. A higher score represents a stronger bond with the therapist. The English version of the questionnaire has been found to be valid. The Hebrew version of the questionnaire has been found to be reliable (α = 0.84) [71].

#### 2.4.11. Home Exercise Adherence

Home exercise adherence assessment was performed using the Exercise Adherence Rating Scale (EARS), which assesses an individual’s adherence to performing exercises which were instructed by healthcare professionals to perform at home. This self-report questionnaire includes six items rated on a Likert-type scale ranging from 0 (*Completely agree*) to 4 (*Completely disagree*) [72]. In one item, the phrase “my healthcare professional” was replaced with “the group instructor” to align with the study. For example: “I do less exercises than recommended by the instructor.” The overall score of the questionnaire is the sum of its items, and the score range is between 0 and 24. A higher score indicates a higher level of adherence to performing exercises at home. The English version of the questionnaire has been found to be reliable (α = 0.81) and valid (a one-factor solution explaining a total of 71% of the variance in adherence to exercise in the exploratory factor analysis.

### 2.5. Procedure

Prior to the intervention, participants completed a self-report questionnaire that gathered information about their demographic status and clinical data. Afterwards, self-report questionnaires were administered to assess the levels of the dependent variables- physical and emotional fall risk factors. Participants were assisted in reading the questions, if necessary, while ensuring uniformity in the way the questions were read and considering the level of vigilance and attentiveness of each participant. This was done to prevent any potential bias among the participants. In addition, objective measurements were conducted to evaluate the participants’ levels of balance. The pre-intervention assessments lasted approximately one hour.

The PTE+DMT group received an intervention that integrated OEP-based techniques and DMT, while the PTE control group received OEP-based techniques only. At the end of the sixth session, participants from both groups were instructed to perform nine exercises independently at home, at least twice a week.

Following the intervention, participants from both groups completed the same series of measurements that were conducted before the intervention. Additionally, each participant was asked to complete a self-report questionnaire to assess the level of the therapist–patient bond. The instructor of the PTE+DMT group administered the questionnaire to the participants of the PTE group, while the instructor of the PTE group administered it to the participants of the PTE+DMT group. This was done to minimize the impact of the questionnaire administrator on the outcome variable. Approximately one month after the end of the intervention, a self-report questionnaire was administered to measure the level of home exercise adherence. The post-intervention assessments lasted approximately one hour.

### 2.6. Data Analysis

Descriptive statistics were calculated for all outcome measures, including median and interquartile ranges. Means and standard deviations were also computed for continuous variables related to objective balance. In order to examine the existence of differences in the outcome variables between the PTE+DMT group and the PTE group and within each group, before and after the intervention, a Fisher’s exact probability test, Wilcoxon test, and Mann/Whitney U test analyses were performed. Furthermore, to compare the percentage changes between groups in the outcome variables, a Mann/Whitney U test was performed.

The ceiling effect of the measurements was measured by calculating the percentage of participants who received the maximum score. The presence of a ceiling effect was defined as when more than 15% of the participants received the maximum score. This indicates that a population with a high level of the trait measured in the measurements may not be evaluable, as items at the top of the scale are missing, and therefore the content validity is limited [73]. All statistical analyses were performed using IBM SPSS Statistics version 27 (IBM Corp., Armonk, NY, USA), and significance was determined at *p* values ≤ 0.05.

In addition, sample size for a future study was calculated using the G*Power software version 3.1.9.7, based on the results of the present study and previous research on the primary variables of balance (measured by TUG) [74,75] and FOF (measured by Short FES-I) [76,77]. The effect size, power, and alpha value were set at 0.8, 0.8, and 0.05, respectively, and a dropout rate of 20% was assumed.

### 2.7. Ethical Issues

All participants provided informed consent prior to any evaluation during the enrolment phase. All participants were provided with written and verbal information about the study. Participants were informed that they had the option to withdraw from the study at any time without penalty or censure. All data collecting and management procedures took the participants’ privacy and confidentiality into account.

## 3. Results

### 3.1. Participants

Data from a total of eight female participants aged 81–91 years (median = 86 [81.25–90.75] years) were analyzed. Table 1 shows the participants’ main characteristics in each group, including age, falls in the last year, use of an assistive device, and whether they live alone. No statistically significant differences were identified between the groups in baseline demographic and clinical variables.

### 3.2. Pre-Test and Post-Test between Groups Differences

As shown in Table 2, before the intervention no significant differences between the groups were found. After the intervention, the PTE+DMT group had a significantly lower 5STS score than the PTE group (U = 0, *p* = 0.025). In addition, the PTE+DMT group had a significantly lower FOF SIQ-score than the PTE group (U = 1.5, *p* = 0.05). No other significant differences between the groups were found in the outcome variables.

### 3.3. Pre-Test and Post-Test within-Groups Differences

**Figure 1:**
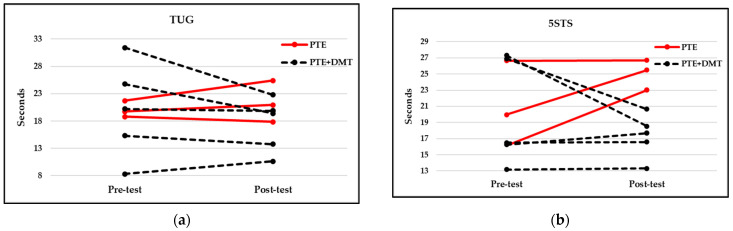
(**a**) Participant scores change of TUG test; (**b**) participant scores change of 5STS test.

As shown in Table 2, no significant within-subjects differences were found in the outcome variables. In addition, Figure 1 illustrates the changes in TUG and 5STS test scores for both groups. The mean change score for TUG was 2.7 and 1.32 s in the PTE+DMT group (pre-test: 20 ± 8.83; post-test: 17.3 ± 4.95) and the PTE group (pre-test: 20.09 ± 1.49; post-test: 21.41 ± 3.82), respectively. The mean change score for 5STS was 2.67 and 4.15 s in the PTE+DMT group (pre-test: 20.02 ± 6.59; post-test: 17.35 ± 2.71) and the PTE group (pre-test: 20.92 ± 5.32; post-test: 25.07 ± 1.88), respectively.

Furthermore, in the PTE+DMT group, 80% of the participants received the maximum score in the therapist–patient bond scale, and most participants (87.5%) of both groups used the upper end of the therapist–patient bond scale.

### 3.4. Comparison of Percentage Changes between Groups

As presented in Table 3, no significant differences were observed between the percentage changes in the groups.

### 3.5. Sample Size for a Future Study

Calculations for future RCT indicated a need for a larger sample size, with a total of 90 participants evenly divided into three groups: PTE+DMT, PTE, and a control group without intervention.

## 4. Discussion

The current study presented a novel fall prevention intervention in older adults that integrates PT exercise and DMT. The current result showed a positive significant effect on balance and muscle strength, as measured by the 5STS score, in the PTE+DMT group compared to the PTE group. These findings align with previous research demonstrating that DMT improves motor function, balance, and gait in older adults with neurodegenerative diseases [78]. Additionally, these findings extend previous studies that have demonstrated that DMT significantly improved diurnal cortisol slope (a physiological outcome) and daily functioning outcomes, in addition to psychological outcomes (e.g., depression), in older adults with mild dementia, compared to physical exercise [41]. These results emphasize the holistic approaches of DMT, based on the empirically supported assertion that mind and body are inseparable and interconnected and that changes in the mind reflect changes in the body and vice versa [39,79].

Additionally, this difference between the groups may be due to the use of different attentional focus during the exercises [80]. It suggests that the PTE+DMT group used an integration of three attentional focus types: (1) external focus (focusing on the effects of movement in the environment) [81], (2) internal focus (focusing on body movements), and (3) holistic focus (focusing on the general feelings or sensations associated with completing a movement) [82]. In contrast, the PTE group may have only used internal focus while participants were instructed to move body parts (e.g., to straighten their legs) without other instructions (e.g., to rise up while paying attention to the contact between their feet and the floor). Previous research has shown that external focus can enhance balance learning in older adults compared to internal focus [83,84], and that holistic focus may be as effective as external focus [85]. However, internal focus can still facilitate learning if combined with external focus during movement execution [86]. Therefore, the improved performance in the 5STS test in the PTE+DMT group, compared to the PTE group, suggests that an integrated focus of attention may enhance motor learning.

No significant difference was found between the groups in TUG, in contrast to the 5STS test, where a significant difference was observed between the groups. This may be due to the fact that only the 5STS test was practiced as an exercise in the intervention. This is aligned with previous findings suggesting that interventions that included exercises which were then measured as objective physical abilities produced significant improvement [87,88]. However, it is noteworthy that the mean change score for TUG in the PTE+DMT group was 2.7 s, which is higher than the following values of TUG: minimal clinically important change (MCID) and the minimal detectable change (MDC) (i.e., the smallest change or difference in an outcome measure that is perceived as beneficial and would lead to a change in the patient’s medical management) [89,90] (1.4 s [74] and 1.8 s [75], respectively, in community-dwelling older adults). In contrast, the mean change score for TUG in the PTE group did not reach the MCID and MDC values (1.32 s).

The current result showed a positive significant effect on FOF, as measured by the FOF SIQ score, in the PTE+DMT group compared to the PTE group. This result is consistent with previous reviews that reported a reduction in FOF levels following body–mind interventions, such as yoga and dance [91,92]. However, no significant changes were found in other measures of falls-related psychological concerns. These results are aligned with a study that conducted a short-term exercise intervention for preventing falls with two sessions per week for 3 months and found physical improvements, but not emotional changes [93]. However, this study demonstrates that even in an intervention targeting emotional aspects, physical changes may be observed with few emotional changes. Additionally, the current study assessed various measures of falls-related psychological concerns. The constructs of FSe, balance confidence and FOF have been reconceptualized, but there is sometimes overlap between the construct measures [94,95]. It is possible that using multiple questionnaires addressing similar contexts and indistinct constructs led to poor self-reporting quality and can be burdensome.

In addition, previous studies have found that there are factors associated with FOF: old age, women, history of falls, living alone [96,97], and using an assistive device [98]. Examining the characteristics data of the current study participants, all of them were above 81 years old, women, 62.5% fell in the past year, 87.5% living alone, and 37.5% using an assistive device. The high prevalence of these factors among the participants and their unchangeable nature may have made it challenging to reduce FOF in a short-term intervention. Furthermore, the present study took place during the fifth and the largest thus far infection wave of the coronavirus 2019 (COVID-19) pandemic [99], and a year after the exit from the third quarantine in Israel. Research has shown that the pandemic and the resulting quarantines have led to an increase in FOF [100], anxiety, fear [101], and a decline in physical and mental health among older adults, which makes them highly vulnerable to the risk of falling upon easing of the quarantine [102]. This may have made it difficult to observe more significant improvements in falls-related psychological concerns in this study, given that the COVID-19 era may have exacerbated these concerns. Future studies should examine the long-term impact of COVID-19 and the decrease in pandemic outbreak impact on these measures.

A trend towards improvement in the physical components of self-perceived health status was observed only in the PTE+DMT group (*p* = 0.08). Similarly, a previous systematic review has reported positive effects of body–mind exercise (body movement combined with mental concentration) on the self-perceived health status in healthy Chinese older adults [103]. Previous research has also linked worse self-perceived health status to a decline in sensory and functional abilities among Spanish older adults [104]. Therefore, it is possible that awareness to the physical-sensory experience and to the success in performing movements among participants in the PTE+DMT group contributed to the improvement tendency in their self-perceived health status. Interestingly, a trend towards a decline was observed in the mental components of self-perceived health status in the PTE+DMT group (*p* = 0.08). It is possible that the participants had a positive bias assessment of their mental health status before the intervention, as found in a meta-analysis where participants evaluated themselves overly positively in self-report questionnaires [105]. Future studies should examine these results using a larger sample size.

The current result of the therapist–patient bond questionnaire after six sessions showed that 80% of the participants in the PTE+DMT group received the maximum score, indicating a ceiling effect, and most participants (87.5%) in both groups used the upper end of the therapist–patient bond scale. These findings are consistent with previous studies that indicate patients often develop a strong bond with their therapist rapidly [106], abruptly, and early in therapy [107]. Additionally, the high prevalence of loneliness among older adults [108] might contribute to their tendency to form satisfying relationships with others, including their therapist [109]. Zilcha-Mano [110], highlights the difference between the “trait-like” component of alliance (i.e., individual baseline characteristics that may be a prerequisite for therapeutic work), and the “state-like” component ( i.e., characteristics that develop over the course of treatment and may change during treatment and facilitate symptomatic change). In addition, patient characteristics such as socially desirable responses may cause a ceiling effect on patient-rated alliance measures [111]. Hence, it is possible that the current results were influenced by the participant’s trait-like parameters (e.g., willingness to form a relationship), and that a longer intervention could develop the state-like component over time in the PTE+DMT group, resulting in changes in other measures such as FOF and home exercise adherence.

Home exercise adherence was assessed one-month post-intervention and was found to be higher in the PTE+DMT group (median = 16 [11,12,13,14,15,16,17,18,19,20]) compared to the PTE group (median = 8 [6,7,8]), although this difference did not reach statistical significance (*p* = 0.095). It is suggested that this higher result in the PTE+DMT group may be attributed to the participants’ trend towards improvement in self-perceived physical health status, as previous research has indicated that health-related physical function is a major factor for exercise adherence [112,113]. Furthermore, older adults who perceived themselves to be physically active were more likely to persist in exercising at home [113]. To validate this suggestion, future studies with larger sample sizes and longer follow-up durations are required.

The present study has several limitations that should be addressed when drawing conclusions for future research. To examine the study protocol, the present study was conducted with a limited sample size and included only six sessions. The statistical analyses are limited due to the small sample size, and, therefore, conclusions should be drawn with caution. In future studies, a larger sample size could enable parametric analysis. In addition, the participants of this study completed a large number of questionnaires, which may have affected the quality of their self-reporting. Additionally, the study population included only women over 80 years of age, most of whom had history of falls and high fall risk. Hence, these generally vulnerable participants require a timely intervention to enhance their physical and emotional measures [93,114,115].

## 5. Conclusions

The current study presented a novel, short-term intervention for preventing falls in older adults that integrates PT exercise and DMT. The findings revealed that this intervention is effective in improving measures of balance and fear of falling, compared to PT exercise alone. However, no other significant differences were found between the groups in terms of fall-related psychological concerns, self-perceived health status, therapist–patient bond, and home exercise adherence. The findings demonstrate the feasibility and potential benefits of an intervention that integrates both physical and emotional aspects to reduce fall risk in older adults, and underline the body–mind connection. Moreover, the findings highlight the importance of research and clinical collaboration between health professionals, which may lead to more holistic intervention strategies.

For future research, the present study suggests several modifications to the research protocol: (1) an intervention with a larger number of sessions; (2) an additional control group without intervention; (3) a larger sample size of a total of 90 participants divided equally into three groups (PTE+DMT, PTE, and a control group without intervention); (4) the utilization of distinct and relevant questionnaires to assess falls-related psychological concerns—FOF SIQ and Short FES-I; (5) incorporating the 5STS test as both a pre-post-test and an intervention exercise to examine the effectiveness of the intervention in a larger research design; (6) including process variables to examine the emotional experience effect (e.g., participants’ subjective vitality) for a more comprehensive understanding of the changes in the outcome variables; and (7) performing parametric statistical analyses.

## Figures and Tables

**Table 1:** Participants’ main characteristics.

Characteristics	PTE+DMT Group (*n* = 5)	PTE Group (*n* = 3)	*p*-Value
Age (years)	90 (81–91)	85 (81–87)	0.45
Falls in the past year (*n*)	2	3	0.18
Assistive device (*n*)	2	1	0.56
Living alone (*n*)	5	2	0.38

Values are presented as median (interquartile range) or *n*. PTE = physical therapy exercises; DMT = dance movement therapy.

**Table 2:** Pre-test and post-test between and within groups differences results expressed by Wilcoxon test and Mann/Whitney U test.

Variable	PTE+DMT Group (*n* = 5)				PTE Group (*n* = 3)				PTE+DMT Group vs. PTE Group			
	Median (IQR)		Z	*p*-Value	Median (IQR)		Z	*p*-Value	Pre-Test		Post-Test	
	Pre-Test	Post-Test			Pre-Test	Post-Test			U	*p*-Value	U	*p*-Value
FRQ	9 (2–11)	9 (4–11)	−0.74	0.46	11 (9–12)	11 (7–14)	0	1	3.5	0.22	4	0.29
Barthel index	95 (85–100)	90 (87.5–97.5)	−0.58	0.56	90 (90–90)	85 (80–90)	−1.34	0.18	4.5	0.34	2.5	0.12
TUG	20.21 (11.81–28.09)	19.21 (12.21–21.34)	−1.21	0.23	19.73 (18.81–21.73)	20.96 (17.84–25.43)	−1.07	0.29	7	0.88	4	0.3
5STS	16.51 (14.71–27.09)	17.68 (14.95–19.59)	−0.41	0.69	19.95 (16.15–26.65)	25.49 (23.02–26.71)	−1.6	0.11	7	0.88	0	0.025 *
FOF SIQ	3 (1.5–4)	3 (2–3.5)	−0.14	0.89	4 (3–4)	4 (4–4)	−1	0.32	4	0.34	1.5	0.05 *
FFQ-R	20 (14.5–22.5)	19 (15–21.5)	−0.69	0.49	21 (20–22)	21 (21–24)	−1.34	0.18	5.5	0.54	2	0.099
Short FES-I	15 (7.5–19.5)	10 (7.5–20.5)	−0.67	0.5	17 (15–17)	20 (18–20)	−1.6	0.11	6.5	0.76	3.5	0.23
FFABQ	27 (6.5–36.5)	18 (3–25)	−0.94	0.35	29 (12–31)	24 (11–33)	−0.54	0.59	5	0.45	4	0.3
ABC	42.81 (33.44–73.28)	60 (46.72–63.91)	−0.73	0.47	45 (45–61.88)	44.38 (40.63–54.38)	−0.54	0.59	5	0.72	2	0.16
PCS-12	28.41 (22.06–46.73)	48.24 (41.01–52.34)	−1.75	0.08	38.14 (26.38–46.26)	30.58 (26.67–50.48)	0	1	7	0.88	4	0.3
MCS-12	47.66 (44.37–56.69)	38.73 (33.35–49.18)	−1.75	0.08	44.57 (34.75–52.47)	38.19 (30.38–42.67)	−1.6	0.11	4	0.3	7	0.88
Therapeutic bond		5 (4.46–5)				4.42 (4.25–4.42)					3	0.15
EARS		16 (11–20)				8 (6–8)					2	0.095

Values are presented as median (interquartile range). Asterisk denotes a significant difference between or within the groups. PTE = physical therapy exercises; DMT = dance movement therapy FRQ = fall risk self-assessment tool; TUG = timed up and go test; 5STS = five times sit to stand; FOF SIQ = fear of falling single item question; FFQ-R = fear of falling questionnaire revised; Short FES-I = short falls efficacy scale-international; FFABQ = fear of falling avoidance-behavior questionnaire; ABC = activities-specific balance confidence scale; PCS-12 = the 12-item short form survey physical component summary; MCS-12 = the 12-item short form survey mental component summary; Therapeutic bond = working alliance inventory-bond subscale; EARS = exercise adherence rating scale.

**Table 3:** Comparison of percentage changes between groups results expressed by Mann/Whitney U test.

Variable	PTE+DMT Group					PTE Group			PTE+DMT Group vs. PTE Group	
	Participant 1	Participant 2	Participant 3	Participant 4	Participant 5	Participant 1	Participant 2	Participant 3	U	*p*-Value
FRQ	0	−18.18%	9.09%	11.11%	0	−22.22%	27.27%	−8.33%	5	0.72
Barthel index	0	6.25%	0	−5.26%	−5%	−5.56%	−11.11%	0	3	0.17
TUG	27.76%	−21.59%	−9.93%	−27.43%	−1.68%	6.24%	−5.18%	17.05%	4	0.3
5STS	1.06%	0.48%	−23.17%	−32.13%	8.73%	42.54%	0.23%	27.77%	3	0.18
FOF SIQ	300%	−66.67%	−25%	−25%	50%	0	33.33%	0	6	0.65
FFQ-R	100%	−5%	−4.76%	−4.17%	−40%	9.09%	5%	0	3	0.18
Short FES-I	228.57%	−57.9%	−10%	−33.33%	−12.5%	17.65%	33.33%	5.88%	3	0.18
FFABQ	275%	−60.87%	−25.93%	−77.78%	−100%	−8.33%	−22.58%	13.79%	3	0.18
ABC	−20.77%	92.31%	18.97%	16.46%	0	−1.39%	−34.34%	20.83%	4	0.48
PCS-12	−8.94%	83.72%	119.93%	73.06%	19.3%	9.13%	−19.81%	1.1%	2	0.1
MCS-12	−27.24%	−35.16%	−9.13%	−22.81%	0.28%	−14.3%	−18.68%	−12.58%	8	0.66

Values are presented as percentage change, which represents the percentage of change in a variable between two time points (pre-test and post-test). PTE = physical therapy exercises; DMT = dance movement therapy. FRQ = fall risk self-assessment tool; TUG = timed up and go test; 5STS = five times sit to stand; FOF SIQ = fear of falling single item question; FFQ-R = fear of falling questionnaire revised; Short FES-I = short falls efficacy scale-international; FFABQ = fear of falling avoidance-behavior questionnaire; ABC = activities-specific balance confidence scale; PCS-12 = the 12-item short form survey physical component summary; MCS-12 = the 12-item short form survey mental component summary; therapeutic bond = working alliance inventory-bond subscale; EARS = exercise adherence rating scale.

## Data Availability

The data are available upon reasonable request.
